# Skunk and Raccoon Rabies in the Eastern United States: Temporal and Spatial Analysis

**DOI:** 10.3201/eid0909.020608

**Published:** 2003-09

**Authors:** Marta A. Guerra, Aaron T. Curns, Charles E. Rupprecht, Cathleen A. Hanlon, John W. Krebs, James E. Childs

**Affiliations:** * Centers for Disease Control and Prevention, Atlanta, Georgia, USA

**Keywords:** Rabies, epizootic, raccoon, skunk, wildlife, zoonosis, spatial analysis, eastern United States

## Abstract

Since 1981, an epizootic of raccoon rabies has spread throughout the eastern United States. A concomitant increase in reported rabies cases in skunks has raised concerns that an independent maintenance cycle of rabies virus in skunks could become established, affecting current strategies of wildlife rabies control programs. Rabies surveillance data from 1981 through 2000 obtained from the health departments of 11 eastern states were used to analyze temporal and spatial characteristics of rabies epizootics in each species. Spatial analysis indicated that epizootics in raccoons and skunks moved in a similar direction from 1990 to 2000. Temporal regression analysis showed that the number of rabid raccoons predicted the number of rabid skunks through time, with a 1-month lag. In areas where the raccoon rabies virus variant is enzootic, spatio-temporal analysis does not provide evidence that this rabies virus variant is currently cycling independently among skunks.

In North America, variants of rabies virus are maintained in the wild by several terrestrial carnivore species, including raccoons, skunks, and a number of bat species. Each antigenically and genetically distinct variant of the virus in mammalian species occurs in geographically discrete areas and is strongly associated with its reservoir species (*1*). Within each area, a spillover of rabies into other species occurs, especially during epizootics (*2*). As a result of spillover, a variant may eventually adapt to a secondary species, which may begin to serve as an alternative reservoir species. This phenomenon of spillover and cross-species adaptation has been inferred from historical relationships (*2*) but is poorly understood and not routinely investigated.

In the late 1970s, an epizootic of raccoon rabies was reported on the Virginia/West Virginia border attributed to the translocation of raccoons from the southeastern United States (*3*). This epizootic has spread northward and southward throughout the eastern United States (Figure 1a,b). The establishment of rabies in this species has raised public health concerns about an increased risk for rabies transmission to the human population because the raccoons are well adapted to living at unusually high densities in urban and suburban environments (*4*,*5*). As a novel potential control method, several states have initiated raccoon vaccination programs using an oral rabies vaccine (*6*–*9*).

**Figure 1:**
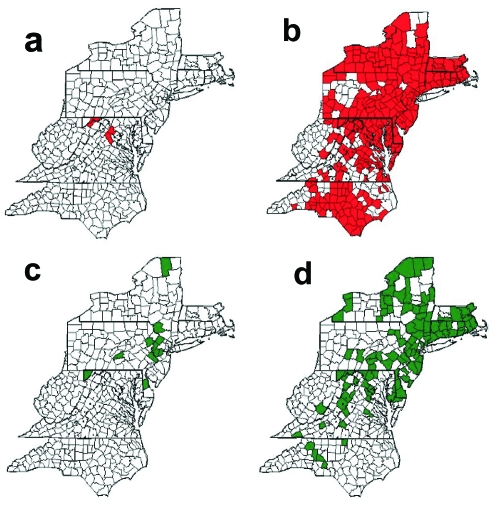
Counties with at least one rabies epizootic among raccoons, 1981(a) through 2000 (b); and among skunks, 1990 (c) through 2000 (d), in the mid-Atlantic states, 1981–2000.

Beginning in 1990, a concomitant increase in the number of cases of skunks infected with the raccoon rabies virus variant has occurred in these states (Figure 1c,d). Additionally, these cases appeared to be preceded by cases in raccoons, both temporally and spatially. Moreover, in a growing number of counties in Massachusetts and Rhode Island, the number of rabid skunks has surpassed the number of rabid raccoons. Whether the increasing number of cases in skunks is a result of spillover from raccoons or the raccoon rabies virus variant has begun to circulate independently within the skunk population remains unclear. The establishment of an independent cycle of rabies in the skunk population may have serious consequences for rabies vaccine baiting programs because the current oral vaccine for raccoons is not as effective in skunks (*10*).

The epizootiology of raccoon rabies in the eastern United States has been investigated in several states, including Virginia (*11*,*12*), Connecticut (*13*), and Maryland (*14*,*15*). Models have been developed to describe the spatial and temporal patterns of raccoon rabies epizootics (*16*–*18*). Several studies have also described the behavior of skunk rabies epizootics in western North America (*19*–*21*), Texas (*22*), and Canada (*23*). The existing raccoon and skunk rabies studies show that epizootic patterns appear to differ between skunks and raccoons, possibly because of differences between the species, rabies virus variants, or environmental factors. However, no documented studies exist on the relatively recent increase of rabies in skunks caused by the raccoon rabies virus variant in the eastern United States. In light of the recent efforts to implement rabies control programs for raccoons in the eastern United States, the epizootiology of raccoon rabies virus variant occurring in skunks in this part of the country needs to be better understood.

The objectives of this study were to describe the epizootiology of skunk rabies in the eastern United States, determine if skunk and raccoon rabies epizootics are associated spatially and temporally, and introduce methods to assess evidence of spillover of rabies from raccoons to skunks compared with independent cycling of the virus within the skunk population.

## Materials and Methods

### Surveillance Data

Rabies case data for raccoons and skunks for each county by month from 11 states (Connecticut, Delaware, Maryland, Massachusetts, New Jersey, New York, North Carolina, Pennsylvania, Rhode Island, Virginia, and West Virginia) were used for analysis. Only counts of rabid animals per county were used because not all counties reported total numbers of animals submitted for testing. The observation period for each county started when the first case of raccoon or skunk rabies was reported, with a maximum study interval of 20 years (1981–2000) and a minimum of 11 years (1990–2000). The lengthy study interval reduced the variability of reporting within a county that may be observed when an epizootic arrives. The unit of analysis was the number of laboratory-confirmed rabies cases in raccoons and skunks reported per month by county. To identify counties that had an appreciable number of skunks infected with rabies, analysis was restricted to counties that reported at least 12 rabid skunks within 12 months of first detecting rabies in skunks. This average of one rabid skunk per month corresponded to the 90th percentile of all counties reporting at least one rabid skunk, and 36 counties met this criterion. Upon examination, one county was excluded because of geographic isolation in the western part of Maryland (Garrett County) that would have severely biased the spatial analysis, and three counties in New York (Clinton, Franklin, and Oswego) were excluded because rabies found in skunks was the result of spillover from a red fox epizootic emerging from Canada (*24*).

### Descriptive Analysis

Rabies epizootics among skunks and raccoons in the 32 counties used for our analyses were identified by using the following algorithm: an epizootic began when the monthly number of rabid animals reported was greater than the county’s monthly median for two consecutive months and ended when this number was less than or equal to the county median for two consecutive months (*16*). Additionally, an epizootic had to be at least 5 months in duration. In calculating a county’s monthly median number of rabid animals, months occurring before the appearance of the first rabid animal were excluded. For example, if rabid skunks first appeared in a county on June 1, 1994, then the months before were excluded for calculation of the skunk median. In that same county, if rabid raccoons appeared on December 1, 1993, then the months before were excluded from calculation of the raccoon median. The size and length of epizootics were compared between species by a Wilcoxon rank sum test. The Kolmogorov-Smirnov two-sample (KS) test was used to assess seasonal differences in the number of rabies cases by species.

### Temporal Analysis

A series of Poisson regression models were used to further explore the relationship between the number of rabid skunks and rabid raccoons. The outcome variable was defined as the log number of rabid skunks. The predictor variables were the number of rabid raccoons, time (continuous 1–140 months), and calendar month of report. The time variables started at 1 with the appearance of the first rabid animal (skunk or raccoon) and continued for up to a total of 140 months (maximum number of months of observation). All counties had at least 72 months of follow-up, and 50% had more than 107 months of follow-up. To smooth each time series, a moving average of the number of rabid animals was calculated on the basis of the present and previous month’s observations for both species and used for subsequent analyses. A time-squared term, an interaction term of time by number of rabid raccoons and indicator variables for county and calendar month of report, was included in the model. The effect of repeated measures by county was controlled in the analysis by using a generalized estimating equation (*25*). Lag periods of 0 to 5 months for the number of rabid raccoons were introduced and assessed to identify any improved fit in the Poisson regression model, as determined by comparing the log likelihood values. The model with the highest (less negative) log likelihood value was chosen as the best fitting model.

### The full Poisson regression model can be represented as

Log (# of skunks) = β_0_ + β_1_(# of raccoons _t-i_) + β_2_(t) + β_3_(t*t) + β_4_(t*# of raccoons _t-i_) + β_j_(county_j_) + β_k_(month_k_) + E where t = time in months (starts at 1 with first appearance of rabid skunk or rabid raccoon in each county and ends with a maximum value of 140) i = 0–5 lag time in months j = 31 indicator variables representing the 32 counties used in the analysis k = 11 indicator variables representing months, with December being the reference group E=residual error.

### Spatial Analysis

**Figure 2:**
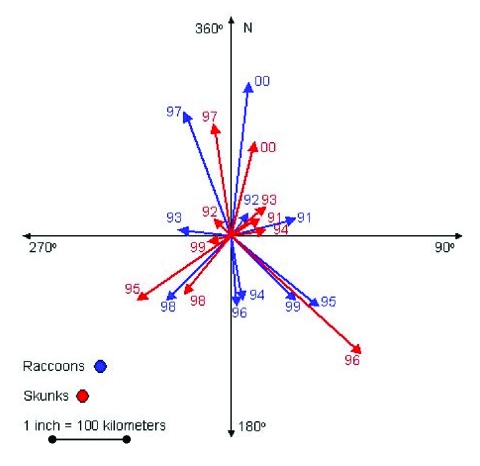
Magnitude and direction (vectors) of successive mean centers of counties from 11 mid-Atlantic states reporting rabies from 1990 to 2000 for raccoons and skunks.

To determine if skunk and raccoon epizootics were associated spatially from 1990 through 2000, the mean center of the counties reporting a rabies case was determined for successive years, 1990–2000, for each species (Crimestat, Department of Justice). The standard deviational ellipse was also calculated, showing the dispersion in two dimensions (Crimestat) of the mean centers. The distance between mean centers of successive years by species was calculated by the Pythagorean theorem. The direction between mean centers was calculated by converting latitudinal and longitudinal coordinates into the Universal Transverse Mercator projections of eastings and northings. On dividing the difference in eastings by the difference in northings, the arctangent was calculated (*26*), yielding the angle (degrees) between the mean centers. The angle was then converted to degrees from the reference angle of 0° (true north). The resulting series of vectors (Figure 2) was used to determine if the mean centers by year for each species were moving in a similar direction. The Watson-Williams test (*27*) was applied to test for a difference in the angle of rotation between the mean centers of each species from 1990 to 2000. An F test was used to determine if the epizootic direction of spread differed between the species. The cumulative mean direction (rotational angle that summarizes a series of vectors through successive years) and circular variance of the mean centers were also calculated (Crimestat).

## Results

### Descriptive Analysis

Of 495 total counties, 344 (69.5%) reported at least one rabid skunk, and 421 (85.1%) reported at least one rabid raccoon (Table 1). The median number of reported rabid raccoons was greater than that for skunks (p<0.0001). Three hundred thirty-nine counties (68.5%) reported rabies in both skunks and raccoons. Within these counties, rabid raccoons preceded rabid skunks in 297 counties (87.6%), rabid skunks preceded rabid raccoons in 30 counties (8.8%), and rabid skunks and raccoons were first reported in the same month in 12 counties (3.5%). The median interval between the initial appearance of rabid raccoons and skunks was 14 months and ranged from –108 months (i.e., rabid skunks preceding rabid raccoons) to 177 months.

**Table 1:** Characteristics of counties within 11 mid-Atlantic states reporting skunk and raccoon rabies, 1990–2000

State	Median no. of rabid animals per county(min, max)		No. of counties with at least one rabid animal (% of total counties in state)	
	Skunks	Raccoons	Skunks	Raccoons
Connecticut	77 (12,209)	380 (225,918)	8 (100)	8 (100)
Delaware	52 (30,97)	269 (168,372)	3 (100)	3 (100)
Massachusetts	80 (14,160)	170 (36,387)	11 (78.6)	11 (78.6)
Maryland	13 (2,82)	226 (105,1075)	23 (95.8)	24 (100)
North Carolina	5 (1,55)	25 (1,166)	41 (41)	86 (100)
New Jersey	31 (4,78)	131 (4,350)	21 (100)	21 (100)
New York	20 (3,202)	127 (2,1294)	55 (88.7)	58 (93.5)
Pennsylvania	11 (1,72)	37 (2,343)	64 (95.5)	66 (98.5)
Rhode Island	56 (9,123)	67 (23,173)	5 (100)	5 (100)
Virginia	7 (1,87)	20 (1,1100)	92 (67.6)	109 (80.1)
West Virginia	8 (1,42)	17 (1,101)	21 (38.2)	30 (54.5)
Total or median	13 (1,209)	42 (1,1294)	344 (69.5)	421 (85.1)

Of 344 counties with at least one reported rabid skunk, 36 counties had at least 12 rabid skunks appearing in the first 12 months after the first rabid skunk appeared. Four counties were omitted for reasons described in the Methods section. In these 32 counties used for more detailed analysis, rabid raccoons preceded skunks in 30 (93.8%) counties, rabid skunks preceded raccoons in 1 (3.1%) county, and both skunks and raccoons appeared in the same month in one county (3.1%). The median interval between the appearance of rabies in raccoons and skunks was 5 months, and ranged from –2 months to 13 months. In the four omitted counties, rabid raccoons preceded skunks in two counties, and rabid skunks preceded raccoons in the remaining two counties.

For all 32 counties, the peak number of rabid raccoons reported was reached by 21 months; the median interval from the first case to the peak number of cases was 10.5 months. In contrast, the interval from the first to the peak number of skunks ranged from 6 to 90 months, with a median interval of 16.5 months (p<0.001). The calendar month when the peak was reached did not exhibit a pattern for rabid raccoons, whereas for rabid skunks there was a strong tendency for the peak to be reached in the last quarter of the year.

Analysis of epizootic characteristics found differences between the first epizootics of each species (Table 2). The first raccoon epizootic was significantly larger (median=126; range 9–494, p<0.0001) than subsequent epizootics among raccoons and also significantly greater than the first skunk epizootic (median=16; range 4–85, p<0.0001). However, after the first epizootic, the epizootics converged and characteristics did not differ, with the exception of the third epizootic, in which the duration and magnitude were lower for skunks than for raccoons. In general, the size of subsequent epizootics among raccoons showed damped oscillations, while skunk epizootics appeared uniform.

**Table 2:** Characteristics of raccoon and skunk epizootics in 32 counties with at least 12 rabid skunks during the first 12 months after the appearance of the first rabid skunk

Characteristic	Epizootic 1		Epizootic 2		Epizootic 3		Epizootic 4		Epizootic 5	
	Raccoon	Skunk	Raccoon	Skunk	Raccoon	Skunk	Raccoon	Skunk	Raccoon	Skunk
No. of counties with epizootics	32	31	22	19	10	12	2	6	0	2
Duration of epizootics-median no. of mon (min,max)	18.5 (6,26)	8 (5,24)^a^	8.5 (5,23)	8 (5,10)	8 (6,12)	6 (5,10)^b^	11.5 (11,12)	8 (5,13)	—	7.5 (5,10)
Size of epizootics-median no. of animals (min,max)	126 (9,494)	16 (4,85)^a^	19 (5,138)	18 (5,39)	19 (9,43)	13 (4,32)^b^	53 (28,78)	18 (6,37)	—	13 (12,14)

^a^p <0.0001. ^b^0.01<p< 0.05.

### Temporal Analysis

**Figure 3:**
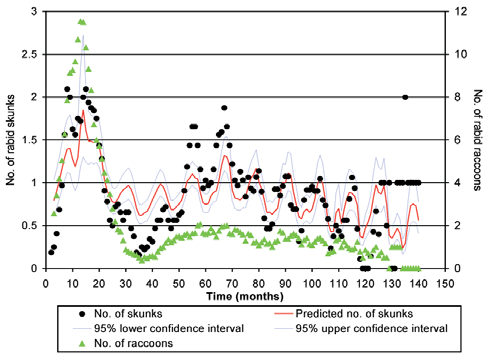
Fitted line resulting from Poisson regression analysis of 32 counties with at least 12 rabid skunks in first 12 months. logY= 0.2835 + 0.0262(RACCOON_t-1_) – 0.0021(time) +0.0020(RACCOON_t-1_*time) + B_i_(county_i_) + B_j_(month_j_)

**Figure 4:**
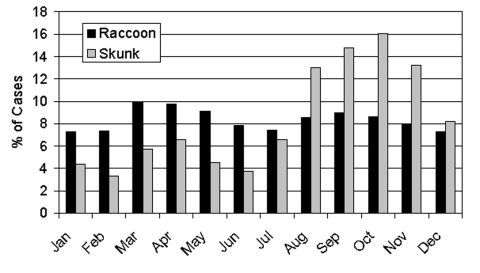
Proportion of rabies cases by month for each species, 1990–2000.

Overall, a significant relationship existed between the number of rabid raccoons (RACCOON) and the number of rabid skunks (SKUNK) (Figure 3, Table 3). Specifically, a significant interaction existed between time and RACCOON on SKUNK with the effect of RACCOON on SKUNK increasing with increasing time. The fit of the models improved significantly when a 1- or 2-month lag for RACCOON was used to predict SKUNK; however, the lag of 1 month provided the best fit. The time-squared term was not significant and was dropped from subsequent models. A month by RACCOON interaction was also tested and did not significantly improve the model fit. At the beginning of the time series, the peak in SKUNK coincided with the larger peak in RACCOON. In the period of approximately 25 months to approximately 50 months, the sharp reduction in RACCOON and coincident reduction in SKUNK was well below model predictions. A second peak in SKUNK at approximately 55 months and 70 months coincided with the increase in RACCOON associated with a second epizootic among raccoons. The model predictions reflected this increase, but the predicted SKUNK fell below the actual SKUNK for the peak months. In addition to a positive correlation with RACCOON over time, SKUNK displayed a strong seasonal component with annual peaks occurring in the fall months (Figure 4). These strong seasonal peaks were unique to skunks, and not present in raccoons (KS 10.6; p<0.0001).

**Table 3:** Summary of Poisson regression analysis of number of rabid raccoons and skunks in 11 mid-Atlantic states

Parameter	Estimate	Standard error	pvalue
Intercept	0.2835	0.1433	0.0479
Raccoon^a^	0.0262	0.0087	0.0025
Time^b^	-0.0021	0.0023	0.3748
Raccoon time	0.002	0.0004	<0.0001
County	^c^	-	-
Month	^d^	-	-

^a^Raccoon = no. of rabid raccoons lagged 1 month ^b^Time = time in months (starting at 1 with appearance of first rabid raccoon or skunk in each county to a maximum value of 140). ^c^Parameter estimates for each county (Worcester, MA, referent group): –0.6529, Berkshire, MA; 0.3297, Bristol, MA; –1.0655, Burlington, NJ; –0.9563, Caroline, MD; –1.1583, Columbia, NY; –1.1351, Dorchester, MD; –0.6038, Dutchess, NY; 0.4234, Essex, MA; –0.2474, Fairfield, CT; –1.0079, Gloucester, NJ; –0.2611, Hartford, CT; –0.3519, Kent, DE; –0.5922, Litchfield, CT; 0.2036, Middlesex, MA; –0.8831, Middlesex, NJ; –1.2675, Monmouth, NJ; –0.5668, Monroe, PA; 0.0172, New Haven, CT; 0.3402, Newport, RI; 0.3887, Norfolk, MA; –0.4269, Orange, NY; –0.0079, Plymouth, MA; 0.3419, Providence, RI; –1.1506, Putnam, RI; –1.0282, Somerset, NJ; –0.3285, Sussex, NJ; –0.3688, Ulster, NY; –1.2583, Union, NJ; –0.3556, Washington, RI; 0.0306, Westchester, NY; –0.6928, Wicomico, MD. ^d^Parameter estimates for each month (December referent group): –0.4074, January; –0.7792, February; –0.6313, March; –0.4830, April; –0.6289, May; –0.8763, June; –0.6548, July; –0.0262, August; 0.2592, September; 0.2851, October; 0.2555, November.

Among the 32 counties used for Poisson regression analysis, four counties in Massachusetts exhibited a general increase in the number of skunks over time (Essex, Middlesex, Norfolk, and Plymouth). A separate model was fit to determine potential differences between these counties and the 28 other counties that did not exhibit a significant increase in rabid skunks over time. The modeling results did not differ among these counties compared with those for the other 28 counties.

### Spatial Analysis

**Figure 5:**
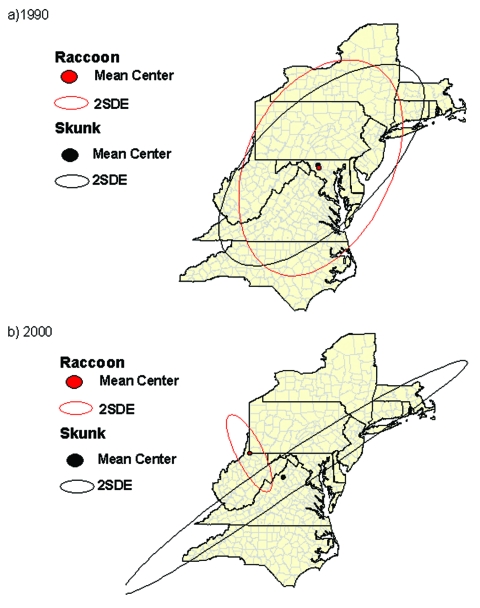
The mean centers and standard deviational ellipses (SDE) of counties reporting rabies in skunks and raccoons in the mid-Atlantic States. a) 1990, b) 2000.

As determined by previously described methods that used vectors (Figure 2), the mean centers of the counties first reporting rabies in both species were in Maryland in 1990 (Figure 5a), and Virginia/West Virginia in 2000 (Figure 5b). The mean direction and distance traveled of the skunk and raccoon epizootics were similar. The mean centers of the epizootics from 1990 to 2000 moved an average of 339.3 km for skunks and 368.2 km for raccoons in a southwesterly direction. Application of the Watson-Williams test resulted in no significant difference between the angles of rotation of successive epizootics [F_1,18_= 0.11(F_1,18;0.95_<4.41, n.s.)], indicating that the mean centers of the skunk and raccoon epizootics were moving in a similar direction. The cumulative mean directions of the epizootics from 1990 to 2000 were 42.06° ± 0.23 for skunks, and 47.76° ± 0.28 for raccoons.

## Discussion

This study examined the relationship between the occurrence of rabies in skunks and raccoons in the eastern United States. The present analysis indicated epizootic cycles of 4–5 years for raccoons and skunks, consistent with previous studies of rabies in raccoons (*16*,*17*) in this region. In comparison, studies of the epidemiology of skunk-variant rabies among skunks from the Midwest reported epizootic cycles with periods ranging from 4 to 5 years (*21*) to 6 to 8 years (*20*,*28*). If raccoon-variant rabies virus becomes established in eastern skunk populations, the periods of epizootic cycles in skunks may subsequently decouple from those of raccoons so that independent cycles among skunks may be observable. However, differences in the periodicity of cycles between these species may be caused by many factors, including differences between the variants, resulting in changes in incubation period, transmission potential, and duration of disease.

The spatial analysis performed in this study indicated that skunk rabies epizootics in the eastern United States are closely coupled to epizootics in raccoons. These epizootics moved in similar directions and traveled similar distances as they progressed upwards along the eastern seaboard. The mean centers of epizootics in each species originated near Maryland and are now situated near the Virginia/West Virginia border as of 2000 (Figure 5a,b). The southwesterly movement is of concern as the raccoon epizootic encroaches areas in the Midwest, where the skunk virus variant predominates.

The Poisson regression analysis showed a statistically significant association between the number of rabid skunks and raccoons through time. The association was weakest during the first months, apparently due to the large number of rabid raccoons that are characteristic of initial rabies epizootics in raccoons (*16*). After the initial peak in numbers at approximately 15 months, both species exhibited a secondary peak at 60 months, consistent with the 4–5 year cycle (*16*,*17*) for raccoon epizootics in the eastern United States. After 80 months, the number of peaks in both species diminished in size and increased in frequency, with the rabies cases in skunks maintaining a strong seasonal component.

The comparison of epizootic characteristics by species also found that the size and duration of epizootics in both species converged after the first epizootic. Of note, however, were the four counties in which rabies cases in skunks were outnumbering those in raccoons near the end of our study period. In these counties, rabies cases in skunks became less sporadic: cases were regularly reported throughout the year but the annual peaks in the fall months remained. As surveillance continues for these four counties, current observations suggest that skunks may be acting as important secondary hosts of the raccoon rabies virus variant in certain geographic areas of the eastern United States and that the potential for independent cycles to emerge exists.

Although we varied the time variable between raccoon and skunk rabies from 0 to 5 months, the best fitting regression model resulted from using a 1-month lag time. This lag time is consistent with the generally accepted incubation period for rabies of 3–8 weeks (*29*), which would permit at least one cycle of virus multiplication among raccoons before transmission from raccoons to skunks. The regression model also showed that reports of rabid raccoons remain fairly constant by month throughout the year. In contrast, the number of rabid skunks showed an independent seasonal pattern that consistently peaked during the fall months (Figure 4). In the Midwest, where rabies is endemic in skunks, the major peak is in late winter and early spring, with a smaller peak in the fall (*20*,*28*). The peaks in winter and late spring have been attributable to the breeding season, and the fall peak to dispersal of juveniles (*23*). Why a dominant fall peak is apparent in the eastern states is not clear at this time. However, during dispersal, skunks may have increased contact with more raccoons, thereby increasing the risk for transmission of rabies. The absence of a spring peak may indicate little to no transmission between skunks in communal winter dens and during the breeding season.

Skunks and raccoons coexist within the same geographic areas in different ecologic niches. Raccoons are social animals that are capable of existing in fairly high densities in close proximity to human habitation and prefer forested habitats (*4*,*5*,*15*). Skunks are rather solitary animals and are found in lower densities than raccoons (*23*). Skunks prefer grasslands (*21*), agricultural areas (*30*), and interfaces between agricultural and nonagricultural lands (*22*). These characteristics would suggest that contact between the two species should occur less frequently than among those of the same species. However, since rabies affects the central nervous system, rabid animals may exhibit aberrant behaviors, leading to increased contact between the species and cross-species transmission of the virus.

Monitoring rabies among skunks in regions where the raccoon rabies virus variant circulates has important implications for public health intervention programs. To control the spread of the raccoon rabies epizootic, an oral rabies vaccine-baiting program has been implemented in several states (*7*–*9*,*31*) after the successful development of a vaccinia virus recombinant vaccine expressing the rabies virus glycoprotein gene (V-RG) for raccoons (*6*,*32*). The oral vaccine has been effective in raccoons. However, as formulated for raccoons, it has not been proven to be as effective for preventing rabies infection in skunks (*10*). Administration of intramuscular rabies vaccines has been shown to be effective in controlling rabies in skunks (*5*), but this method is labor-intensive and cost-prohibitive. The emergence of independent maintenance or cycling of raccoon-associated rabies virus within skunks would necessitate the development of alternative strategies to control rabies within wildlife populations. At least one vaccine candidate (*33*) designed for skunks has been identified but will require further development for this species and prevent spillover of rabies back into the raccoon population.

Currently, we have no evidence that the raccoon rabies virus variant is cycling independently in the skunk population of the eastern United States or that the variant has undergone any genetic adaptations among skunks. However, epizootic rabies in skunks was first reported in 1990 and, with expected epizootics cycling every 4–5 to 6–8 years, it may be too soon to detect decoupling of rabies cycles in skunks and raccoons. Surveillance and monitoring must continue through several cycles to further evaluate additional epizootics for changes in patterns. Additionally, scant information exists on the population densities and behavior patterns of skunks and raccoons in the eastern United States. Field investigations to assess the incidence of rabies in wildlife populations have rarely been conducted. Further research is needed to evaluate environmental factors that can affect the population density and structure, the behavior of both raccoons and skunks, and factors influencing interactions between them. Finally, the genetics of the raccoon rabies virus variant should be monitored for changes that might indicate cross-species adaptation after spillover into skunks. Assessment of these changes and continued surveillance can provide important guidelines to ensure the success of oral rabies vaccination programs for the control of rabies in wildlife and to decrease the risk of acquiring rabies among the human and domestic animal populations.
